# Composite dietary antioxidant index and HPV infection from single and mixed associations to SHAP-interpreted machine learning predictions

**DOI:** 10.3389/fnut.2025.1619742

**Published:** 2025-07-31

**Authors:** Pei Zhang

**Affiliations:** Department of Gynaecology, The First Affiliated Hospital, and College of Clinical Medicine of Henan University of Science and Technology, Luoyang, China

**Keywords:** composite dietary antioxidant index, HPV infection, machine learning, SHAP, WQS, BKMR, NHANES

## Abstract

**Background:**

Some studies have shown that dietary antioxidants may prevent the occurrence of Human Papillomavirus (HPV) infection. However, the relationship between the composite dietary antioxidant index (CDAI) and HPV infection among adult women in the United States remains unknown.

**Methods:**

Participants from the National Health and Nutrition Examination Survey (NHANES) during 2003–2016 were included. Multivariable logistic regression, restricted cubic spline (RCS) regression, weighted quantile sum (WQS) regression, and Bayesian kernel machine regression (BKMR) were used to analyze the associations between CDAI and its sub-components and HPV infection. In addition, nine machine learning (ML) methods were employed to construct predictive models, and SHapley Additive exPlanations (SHAP) was used to further interpret the optimal model.

**Results:**

This study enrolled 9,224 adult female participants. After adjusting for multiple confounding variables, CDAI was independently negatively associated with HPV infection (OR: 0.98, 95%CI: 0.97–0.99, *p* = 0.01). RCS indicated an L-shaped association between CDAI and HPV infection. In the WQS model, the WQS index of CDAI was still robustly negatively associated with HPV infection (OR: 0.78, 95%CI: 0.71–0.86, *p* < 0.0001). In the mixture effect, BKMR analysis confirmed the negative association between six antioxidants and HPV infection. Both WQS and BKMR confirmed that vitamin E had the strongest negative association with HPV infection. Additionally, among the nine machine—learning models, the Gradient Boosting Machine (GBM) showed the best predictive performance [area under curve (AUC) = 0.685]. SHAP analysis indicated that marital status, smoking, drinking, race, age, and CDAI had a significant impact on the model’s prediction.

**Conclusion:**

Antioxidant—rich diets, especially increased intake of vitamin E, are significantly negatively associated with HPV infection. A GBM model with 12 features can effectively predict the occurrence of HPV infection, among which CDAI is an important factor in the model.

## 1 Introduction

Human papillomavirus (HPV) infection is a serious public health issue, which is closely related to a variety of benign and malignant diseases. As a double-stranded circular DNA virus, HPV has an epitheliotropic nature and is capable of infecting the epithelial cells of human skin and mucosa (1). Persistent infection with high-risk types of HPV can lead to malignant tumors such as cervical cancer, anal cancer, and oropharyngeal cancer. Among them, cervical cancer is the most well-known HPV-related cancer (2). According to the global cancer statistics in 2020, it is estimated that worldwide, there are approximately 604,000 new cases of cervical cancer and 342,000 deaths from cervical cancer each year (3). From an epidemiological perspective, HPV infection is highly prevalent and widespread globally. There are significant differences in the infection rates among populations of different regions, ages, and genders (4). Given the huge disease burden caused by HPV infection and its epidemiological characteristics, exploring effective prevention and treatment strategies is of great scientific significance and social value.

Although HPV is a known risk factor for cervical cancer, studies have shown that some proactive measures can prevent the development of HPV infection into cervical cancer (5). In this regard, diet may have considerable potential. On the one hand, a reasonable dietary structure can provide adequate nutritional support for the immune system, helping the body to more effectively recognize and eliminate HPV viruses (6). On the other hand, unhealthy eating habits may increase the risk of HPV infection or promote the progression of the disease (7). A long-term dietary pattern high in sugar, fat, and salt is likely to lead to metabolic disorders in the body, causing problems such as obesity and insulin resistance, which in turn weakens the function of the immune system and reduces the body’s defense against HPV (8, 9).

Oxidative stress refers to a pathological state in which, when the body is stimulated by various internal and external factors, there is an imbalance between the production of reactive oxygen species (ROS) in the body and the antioxidant defense system, leading to excessive accumulation of ROS within cells (10). Studies have shown that there is a close association between oxidative stress and human HPV infection, and this relationship plays an important role in viral infection, persistence, and disease progression (11). However, the existing evidence regarding the association between HPV infection and antioxidant diet is not yet convincing (12). Huang et al. (13) found in a large cross-sectional study that there was a U-shaped association between dietary vitamin A and the risk of HPV, which means that both too low and too high levels of vitamin A are risk factors for HPV. Zheng et al. (14) also found a U-shaped association between vitamin C and the risk of HPV. Vitamin E seems to be a more promising protective factor. Research has shown that there is a negative linear relationship between dietary vitamin E intake and both high-risk and low-risk HPV infections (15). A cohort study from Brazil showed that the serum vitamin E level may have a protective effect against the persistence of non-carcinogenic HPV (16). A multicenter study showed that women in the highest quartile of plasma cis- and total *β*-carotene had approximately half the risk of HPV infection (17). However, Siegel et al. (16) did not find a strong protective effect of serum antioxidant nutrients other than vitamin E measured during four clinical visits on the persistence of type-specific carcinogenic HPV. A randomized controlled trial (RCT) involving 80 subjects showed that oral zinc sulfate supplementation for 3 months could increase the clearance rate of HPV and the regression rate of pre-existing cervical lesions (18). A study from western China showed that a serum selenium concentration exceeding 0.02 mg/kg is a protective factor against HPV infection (19). These studies not only show the exciting potential of antioxidants in the prevention of HPV infection but also present elusive conclusions. It is necessary to comprehensively consider multiple antioxidant nutrients and recognize the interference among nutrients and the potential mixed effects.

The Composite Dietary Antioxidant Index (CDAI) is a comprehensive scoring system used to quantify the overall antioxidant capacity of the diet. This index is evaluated by integrating six key dietary antioxidant components, including six nutrients: vitamin A, vitamin C, vitamin E, zinc, selenium, and carotenoids, thus comprehensively reflecting the antioxidant characteristics of the diet (20, 21). Currently, only one study from Italy has reported the relationship between CDAI and HPV infection. In this study, it was found that women with a high CDAI had a lower probability of being HPV-positive than those with a low CDAI (5). However, considering the differences in populations and the relatively small sample size, it is necessary to explore the relationship between CDAI and HPV infection in the entire American population.

The aim of this study is to explore the single and combined associations between CDAI and its six sub-components and HPV infection in the National Health and Nutrition Examination Survey (NHANES) database. Additionally, this study attempts to construct a predictive model using machine learning and determine the important factors for predicting HPV infection through SHapley Additive exPlanation (SHAP).

## 2 Methods

### 2.1 Study population

The data of this study were derived from seven NHANES cycles from 2003 to 2016. Initially, 71,058 participants were included. Based on the research objectives and design requirements, first of all, since the study focused on a specific adult population, 31,837 individuals under the age of 20 were excluded. Secondly, considering that the study targeted a specific gender category, 18,966 male participants were removed. Furthermore, taking into account the crucial role of the integrity of key variables such as CDAI and HPV infection status in the accuracy of the study, as well as the potential confounding effects brought about by pregnancy factors, 2,212 participants with missing CDAI data, 7,101 participants with unavailable HPV infection status data, and 601 pregnant women were further excluded. Finally, to ensure the effectiveness of statistical analysis, covariates were strictly screened, and a total of 1,121 participants with missing data related to marital status, alcohol consumption status, smoking status, body mass index, cancer, liver problems, hypertension, diabetes, chronic kidney disease (CKD), etc. were excluded. After the above series of rigorous inclusion and exclusion processes, the data of 9,224 participants were finally determined for subsequent research and analysis (Supplementary Figure 1).

### 2.2 Composite dietary antioxidant index

CDAI covers six antioxidant components, namely vitamin A, vitamin C, vitamin E, zinc, selenium, and carotenoids (20, 21). Given the differences in the measurement units of various antioxidants, standardization processing needs to be carried out first. Specifically, the standardization is achieved by subtracting the average intake of the population from the individual nutrient intake and then dividing it by the standard deviation of that nutrient. After standardizing each antioxidant, the standardized values are summed up to obtain the CDAI. Its calculation formula is CDAI= $\overset{n=6}{\underset{i=1}{\sum }}\left(\mathrm{Individual intake}-\mathrm{Mean}\right)/\mathrm{SD}$ . It should be emphasized that the individual dietary nutrient intake is the average value of the intakes obtained from two 24-h dietary recalls. Individuals who only provided the reported values for a single day were not included in this study.

### 2.3 HPV infection

In the NHANES survey, human papillomavirus (HPV) testing was carried out on subjects aged 18 to 59 years old. The testing was conducted in the laboratory relying on the HPV L1 universal primer polymerase chain reaction (PCR) technology. Among them, the biotinylated PGMY09/11 primers were used for detection, and the *β*-globin primers were used as a control. This method can detect 37 types of HPV, including types 6, 11, 16, 18, 26, 31, 33, 35, 39, 40, 42, 45, 51, 52, 53, 54, 55, 56, 58, 59, 61, 62, 64, 66, 67, 68, 69, 70, 71, 72, 73, 81, 82, 83, 84, 89, and IS39. As long as any one of the 37 types is positive, it is defined as a positive HPV infection; otherwise, it is negative (22).

### 2.4 Covariates

Based on a large number of previous literatures, we have determined that the following factors may be confounding factors affecting the association between CDAI and HPV infection (13, 23). Continuous variables include age and daily dietary energy. Categorical variables include race, educational level, marital status, poverty-income ratio (PIR), alcohol consumption, smoking, exercise, body mass index (BMI), cancer, diabetes, hypertension, cardiovascular disease (CVD), chronic kidney disease (CKD), liver disease, and hyperlipidemia. The specific classification is shown in Supplementary Table 1.

### 2.5 Statistical analysis

In the correlation analysis, first, the *t*-test, Mann–Whitney U test, and chi-square test were used to compare the differences in the general characteristics of the participants between the groups with and without HPV infection. Since the outcome was a binary variable, a multivariate logistic regression model was constructed. The logistic regression model adopted multiple model adjustment strategies to verify the robustness of the association. In Model 0, we did not adjust for any confounding factors; in Model 1, we only adjusted for demographic factors; in Model 2, we further adjusted variables such as lifestyle; and Model 3 was a fully adjusted model, which, on the basis of Model 2, further adjusted for a number of comorbidities. Considering that CDAI was a continuous variable, this study also explored its dose–response relationship with HPV infection through restricted cubic spline (RCS) regression. In the RCS model, the Wald test was used to determine whether there was a nonlinear association. In addition, this study also investigated the relationship between CDAI and HPV infection in specific populations, and the likelihood ratio test was used to determine the significance of the interaction. Finally, we conducted several sensitivity analyses. As several potential confounders were missing in individual years, such as condom use data available only in six cycles from 2005–2016, number of sexual partners asked in four cycles from 2009–2016, and HPV vaccination status provided in five cycles from 2007–2016, we additionally adjusted for these variables in sensitivity analyses. Specifically, Sensitivity Analysis 1 included additional adjustments for condom use and number of sexual partners, Sensitivity Analysis 2 added adjustments for HPV vaccination status, and Sensitivity Analysis 3 simultaneously adjusted for condom use, number of sexual partners, and HPV vaccination status.

In order to determine the independent and combined effects of different antioxidants, this study also conducted weighted quantile sum (WQS) regression analysis and Bayesian kernel machine regression (BKMR) analysis. WQS regression is a statistical method used to evaluate the comprehensive effects of multiple exposure factors, especially suitable for handling situations with multiple related exposure factors. In this study, we applied WQS regression to analyze the comprehensive impact of six dietary antioxidants on HPV infection and identify the factor with the strongest influence. In the WQS model, we constructed the WQS index using the quartiles of the intakes of dietary antioxidants, and incorporated the estimated weights of each component into this index. We assumed that the direction of the effect was negative, and randomly divided the data into a training dataset and a validation dataset according to the ratio of 40 and 60%. To obtain robust estimation results, we adopted the bootstrap sampling method with *N* = 1,000 times. BKMR is a non-parametric statistical method that can flexibly capture the mixed effects of multiple exposure factors on the outcome. In the application of the BKMR model, we used the Markov Chain Monte Carlo (MCMC) method to perform up to 20,000 iterations for each BKMR model.

We also constructed a predictive model. Specifically, the 9,224 observed subjects were randomly divided in a ratio of 7:3, resulting in a training set containing 6,458 samples and a test set containing 2,766 samples. First, the Boruta algorithm was used to reduce the dimensionality of the training set and screen out the features that were truly relevant to the target variable. Subsequently, we constructed nine machine learning algorithms to train and build a predictive model for predicting the occurrence of HPV infection. The nine algorithms are Logistic Regression (Logistic), Support Vector Machines (SVM), Gradient Boosting Machine (GBM), Artificial Neural Network, eXtreme Gradient Boosting (Xgboost), K-Nearest Neighbors (KNN), Adaptive Boosting (Adaboost), Light Gradient Boosting Machine (LightGBM), and Categorical Boosting (CatBoost). To ensure the robustness of the model performance, we employed 10-fold cross-validation when training all nine models. We also further evaluated the reliability and clinical applicability of the model through calibration curves and decision curve analysis (DCA) to determine its net benefit. The optimal model was determined by the receiver operating characteristic curve (ROC) and its Area Under Curve (AUC).

To enhance the interpretability of the model, we selected the SHapley Additive exPlanation (SHAP) algorithm for analysis (24). This algorithm calculates the corresponding SHAP value for each feature variable, and this value can accurately measure the contribution of each variable to the model’s output result. By calculating and visualizing the average absolute SHAP values of each feature, we can clearly rank the importance of the variables in the model, enabling us to understand the specific impact of each variable on the prediction result. The SHAP summary plot intuitively presents the comprehensive effect of all features on the model’s prediction, allowing us to quickly identify the key influencing factors. SHAP force plot aims to clearly show, for a single sample, how each feature contributes to the change in the final predicted value.

## 3 Results

### 3.1 Population characteristics

After inclusion and exclusion, there were a total of 9,224 participants with complete data (Supplementary Figure 1). As shown in Table 1, the median age of all participants was 40 years old. There were 5,135 people in the non-HPV infection group and 4,089 people in the HPV infection group. Compared with the non-HPV infection group, the participants in the HPV infection group were younger. In terms of ethnic distribution, the proportion of non-Hispanic White people was lower, and the proportion of African Americans was higher. In terms of educational attainment, the proportion of those with an education level below university was higher. Regarding marital status, the proportions of those who had never been married and those who were divorced/separated/widowed were higher, while the proportion of those who were married/living with a partner was lower. The proportion of people with an income below 1.3 times the poverty line was higher. The proportion of those who never drank alcohol was lower, and the proportion of current drinkers was higher. The proportion of those who never smoked was lower, and the proportion of current smokers was higher. In terms of health indicators, the mean value of CDAI was lower, and the average levels of dietary vitamin A, C, E, zinc, selenium, and carotenoids were also lower. The prevalence rates of hypertension and CVD were higher. However, there were no significant differences between the two groups in terms of daily energy intake, BMI, cancer, diabetes, CKD, liver problems, and hyperlipidemia.

**Table 1:** Characteristics of eligible participants.

Characteristics	Total (*n* = 9,224)	Non-HPV (*n* = 5,135)	HPV (*n* = 4,089)	*P*
CDAI, Median [mean (SD)]	0.52 (4.08)	0.71 (4.05)	0.28 (4.09)	<0.001
Vitamin A [mean (SD)]	546.90 (565.44)	570.68 (588.06)	517.03 (534.26)	<0.001
Vitamin C [mean (SD)]	78.29 (90.46)	80.00 (86.55)	76.14 (95.10)	0.042
Vitamin E [mean (SD)]	7.35 (5.78)	7.58 (6.02)	7.05 (5.45)	<0.001
Zinc [mean (SD)]	9.78 (5.93)	9.92 (6.10)	9.61 (5.71)	0.014
Selenium [mean (SD)]	97.96 (52.54)	99.09 (52.23)	96.54 (52.91)	0.02
Carotenoid [mean (SD)]	8635.97 (11658.71)	9002.42 (11579.50)	8175.79 (11742.65)	0.001
Age (years), Median (Q1, Q3)	40 (30, 49)	41 (31, 50)	39 (28, 48)	< 0.001
Race, n (%)				< 0.001
Non-Hispanic White people	3,880 (42)	2,297 (45)	1,583 (39)	
Mexican American	1,615 (18)	983 (19)	632 (15)	
Non-Hispanic Black	2003 (22)	804 (16)	1,199 (29)	
Others	1726 (19)	1,051 (20)	675 (17)	
Education level, n (%)				< 0.001
Less than college	3,769 (41)	1945 (38)	1824 (45)	
College or higher	5,455 (59)	3,190 (62)	2,265 (55)	
Marital status, n (%)				< 0.001
Never married	2,119 (23)	954 (19)	1,165 (28)	
Divorced/separated/widowed	1724 (19)	710 (14)	1,014 (25)	
Married/living with a partner	5,381 (58)	3,471 (68)	1910 (47)	
Poverty, n (%)				< 0.001
<1.3	2,843 (31)	1,356 (26)	1,487 (36)	
1.3–3.5	3,027 (33)	1,690 (33)	1,337 (33)	
>3.5	2,781 (30)	1788 (35)	993 (24)	
Unkonwn	573 (6)	301 (6)	272 (7)	
Alcohol intake, n (%)				< 0.001
Never drinker	1,511 (16)	995 (19)	516 (13)	
Former drinker	1,254 (14)	697 (14)	557 (14)	
Current drinker	6,459 (70)	3,443 (67)	3,016 (74)	
Day’s energy intake, median (Q1, Q3)	1792 (1,356, 2,299)	1795 (1362.5, 2,297)	1790 (1,345, 2,300)	0.983
Smoking status, n (%)				< 0.001
Never smoker	5,807 (63)	3,513 (68)	2,294 (56)	
Former smoker	1,376 (15)	800 (16)	576 (14)	
Current smoker	2041 (22)	822 (16)	1,219 (30)	
Physical activity (MET, minutes/week), n (%)				< 0.001
<700	2,300 (25)	1,299 (25)	1,001 (24)	
700–2,400	2,221 (24)	1,306 (25)	915 (22)	
≥ 2,400	2,469 (27)	1,341 (26)	1,128 (28)	
Unkonwn	2,234 (24)	1,189 (23)	1,045 (26)	
Body mass index (kg/m^2^), n (%)				0.085
<25	2,991 (32)	1704 (33)	1,287 (31)	
≥25	6,233 (68)	3,431 (67)	2,802 (69)	
Cancer, n (%)				0.17
No	8,713 (94)	4,866 (95)	3,847 (94)	
Yes	511 (6)	269 (5)	242 (6)	
DM, n (%)				0.457
No	8,244 (89)	4,578 (89)	3,666 (90)	
Yes	980 (11)	557 (11)	423 (10)	
Hypertension, n (%)				0.022
No	6,756 (73)	3,810 (74)	2,946 (72)	
Yes	2,468 (27)	1,325 (26)	1,143 (28)	
CVD, n (%)				< 0.001
No	8,825 (96)	4,952 (96)	3,873 (95)	
Yes	399 (4)	183 (4)	216 (5)	
CKD, n (%)				0.314
No	8,279 (90)	4,624 (90)	3,655 (89)	
Yes	945 (10)	511 (10)	434 (11)	
Liver problem, n (%)				0.994
No	8,923 (97)	4,968 (97)	3,955 (97)	
Yes	301 (3)	167 (3)	134 (3)	
Hyperlipidemia, n (%)				0.807
No	3,113 (34)	1727 (34)	1,386 (34)	
Yes	6,111 (66)	3,408 (66)	2,703 (66)	

CDAI, composite dietary antioxidant index; HPV, human papillomavirus; BMI, body mass index; CVD, cardiovascular disease; CKD, chronic kidney disease; DM, diabetes mellitus; SD, standard deviation.

### 3.2 The association of CDAI with HPV infection

Table 2 shows the results of the logistic regression analysis of the association between CDAI and HPV infection. After CDAI was included in the model as the original continuous value, from Model 0 to Model 3, all demonstrated a stable negative association between CDAI and HPV infection. Moreover, in the fully adjusted Model 3, an increase of one unit in CDAI was associated with a 2% decrease in the likelihood of HPV infection [odds ratio (OR): 0.98, 95% confidence interval (CI): 0.97–0.99, *p* = 0.01]. After classifying CDAI according to quartiles, taking quartile 1 as the reference group, in all models (Model 0 to Model 3), the likelihood of HPV infection in the quartile 3–4 groups decreased significantly. In the fully adjusted Model 3, compared with the quartile 1 group, the prevalence of HPV infection in the quartile 3 group (OR: 0.78, 95%CI: 0.68–0.89, *p* < 0.001) and the quartile 4 group (OR: 0.77, 95%CI: 0.66–0.91, *p* = 0.001) decreased by 22 and 23%, respectively.

**Table 2:** The association between CDAI and HPV infection in Multivariate logistic regression.

Characteristics	Model 0		Model 1		Model 2		Model 3	
	OR (95% CI)	*P*	OR (95% CI)	*P*	OR (95% CI)	*P*	OR (95% CI)	*P*
CDAI (continuous)	0.97 (0.96, 0.98)	<0.0001	0.98 (0.97, 0.99)	0.002	0.98 (0.97, 0.99)	0.01	0.98 (0.97, 0.99)	0.01
CDAI (classify)								
Quartile 1	Ref		Ref		Ref		Ref	
Quartile 2	0.83 (0.74, 0.94)	0.002	0.92 (0.82, 1.04)	0.18	0.90 (0.80, 1.03)	0.12	0.91 (0.80, 1.03)	0.14
Quartile 3	0.69 (0.61, 0.77)	<0.0001	0.79 (0.70, 0.89)	<0.001	0.77 (0.67, 0.88)	<0.001	0.78 (0.68, 0.89)	<0.001
Quartile 4	0.71 (0.63, 0.80)	<0.0001	0.82 (0.72, 0.92)	0.001	0.77 (0.66, 0.90)	0.001	0.77 (0.66, 0.91)	0.001

Model 0: No covariate was adjusted. Model 1: Adjusted for age, race, education attainment, marital status, and poverty-income ratio. Model 2: Further adjusted for smoking, drinking status, BMI, physical activity, and energy intake based on Model 1. Model 3: Further adjusted for hyperlipidemia, hypertension, DM, liver diseases, cancer, CVD, and CKD.

**Figure 1:**
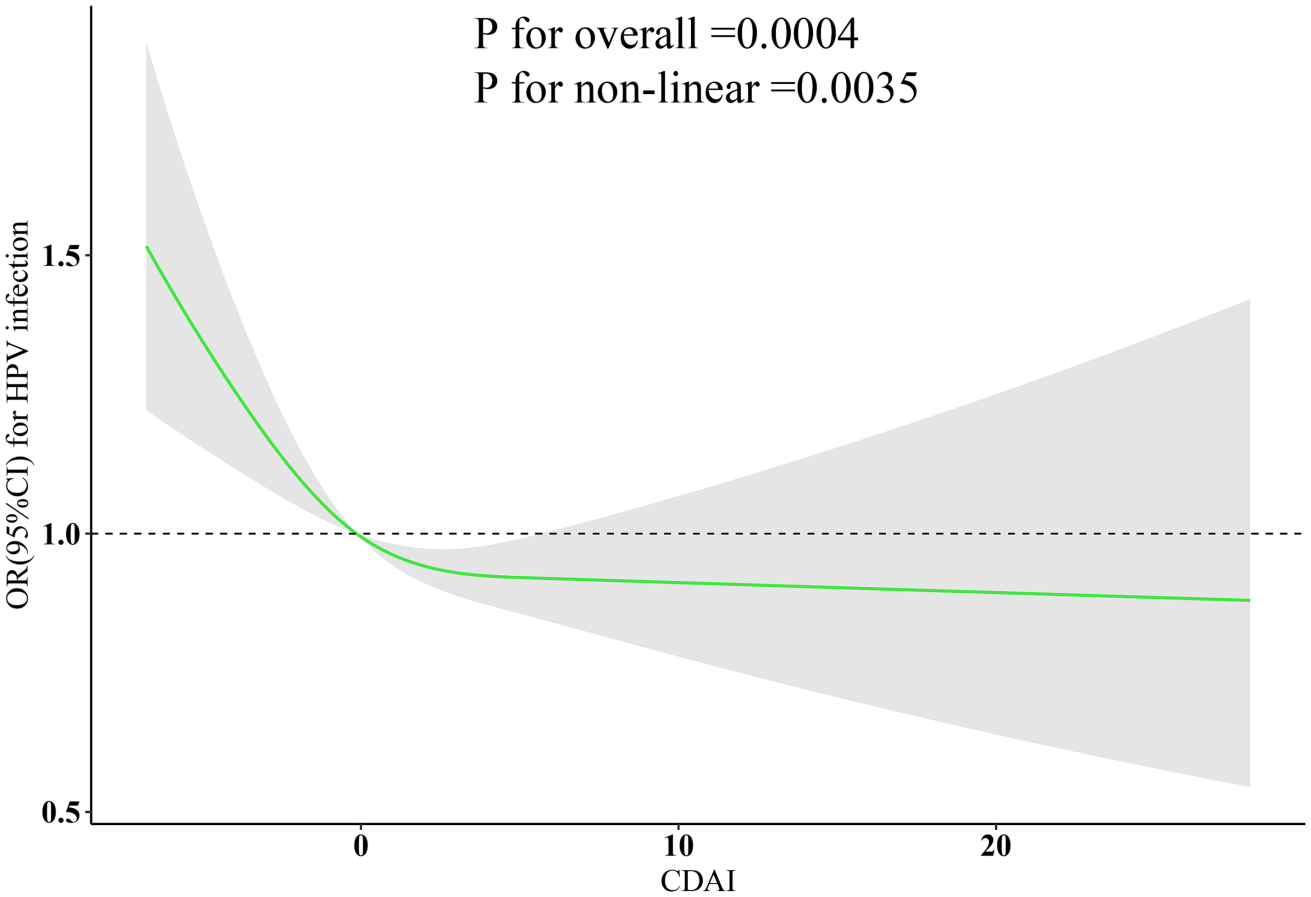
Dose–response relationship between CDAI and HPV infection. Model were adjusted for age, race, education attainment, marital status, poverty-income ratio, smoking, drinking status, BMI, energy intake, physical activity, hyperlipidemia, hypertension, DM, cancer, CVD, CKD, liver problem. CDAI, composite dietary antioxidant index; HPV, human papillomavirus; BMI, body mass index; CVD, cardiovascular disease; CKD, chronic kidney disease; DM, diabetes mellitus; OR, odds ratio; CI, confidence interval.

Figure 1 shows an L-shaped dose–response relationship between the continuous CDAI values and HPV infection. As shown in the figure, when CDAI is less than 0, with the increase of CDAI, the likelihood of HPV infection drops sharply. After that, further increases in CDAI hardly reduce the likelihood of HPV infection.

Supplementary Figure 2 shows the results of the subgroup analysis. The results indicate that being married or cohabiting significantly interacts with CDAI, suggesting that this group of people benefits more from dietary antioxidants (interaction *p* = 0.003).

Supplementary Table 2 shows the results of sensitivity analyses. In Sensitivity Analysis 1, the number of sexual partners and condom use were additionally adjusted for; in Sensitivity Analysis 2, HPV vaccination status was additionally considered; and in Sensitivity Analysis 3, the number of sexual partners, condom use, and HPV vaccination were adjusted for. The results of several sensitivity analyses were consistent with those of the main analysis, further enhancing the reliability of this study.

### 3.3 WQS regression analysis of the individual and overall associations between six antioxidant nutrients and HPV infection

**Figure 2:**
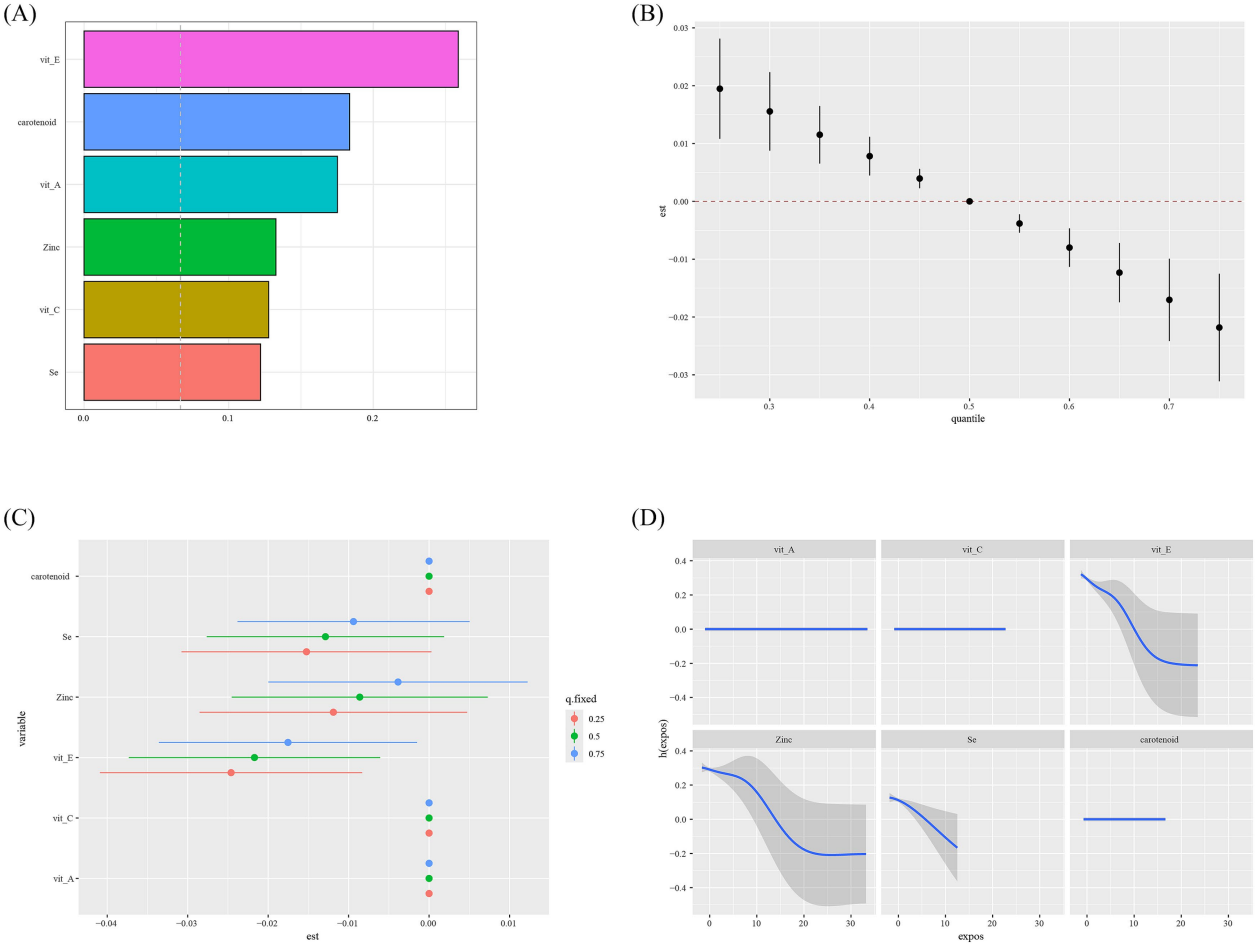
Single and mixed association between CDAI and HPV infection in BKMR and WQS model. **(A)** Weights of WQS index of six dietary antioxidants in HPV infection in WQS model. The dashed black lines represent the cutoff to discriminate which element has a significant weight. **(B)** Mixed association between CDAI and HPV infection in BKMR model. **(C)** Single-exposure effects of individual dietary antioxidants on HPV infection in BKMR model. **(D)** Single exposure dose relationship of individual dietary antioxidants with HPV infection in BKMR model. All models were adjusted for age, race, education attainment, marital status, poverty-income ratio, smoking, drinking status, BMI, energy intake, physical activity, hyperlipidemia, hypertension, DM, cancer, CVD, CKD, liver problem. CDAI, composite dietary antioxidant index; HPV, human papillomavirus; WQS, weighted quantile sum; BKMR, Bayesian kernel machine regression; BMI, body mass index; CVD, cardiovascular disease; CKD, chronic kidney disease; DM, diabetes mellitus.

In the WQS model, the WQS index of the combined antioxidant diet was negatively associated with HPV infection [OR: 0.78; 95%CI: (0.71, 0.86), *p* < 0.0001] (Supplementary Table 3). Additionally, the weights of all six antioxidants in the WQS model exceeded the threshold, with vitamin E having the highest weight of 0.26 (Figure 2A).

### 3.4 BKMR model analysis of the relationships between individual and combined antioxidant nutrients and HPV infection

In the BKMR model, the effects of individual and combined antioxidant nutrients on HPV infection were further verified. Supplementary Table 4 shows the Posterior Inclusion Probability (PIP) values of the relationships between six dietary antioxidants and HPV infection. Among them, vitamin E contributed the most to HPV infection (PIP = 0.68).

Figure 2B shows the cumulative impact on HPV infection when the intakes of all antioxidants are at the same certain percentile, compared with the situation when all elements are fixed at the median. We observed that this result was significant regardless of whether it was at the 25th, 30th, 35th, 40th, 45th, 55th, 60th, 65th, 70th, or 75th percentile. Moreover, as the percentile value increases, the likelihood of HPV infection decreases, indicating that the combination of the six antioxidants reduces the likelihood of HPV infection. In addition, when the remaining five nutrients were fixed at the 25th, 50th, and 75th percentiles respectively, it was found that only vitamin E had a significant negative correlation with HPV infection (Figure 2C). It should be noted that Figure 2D shows that there is also an L-shaped association between vitamin E and HPV infection.

### 3.5 Development of predictive models

**Figure 3:**
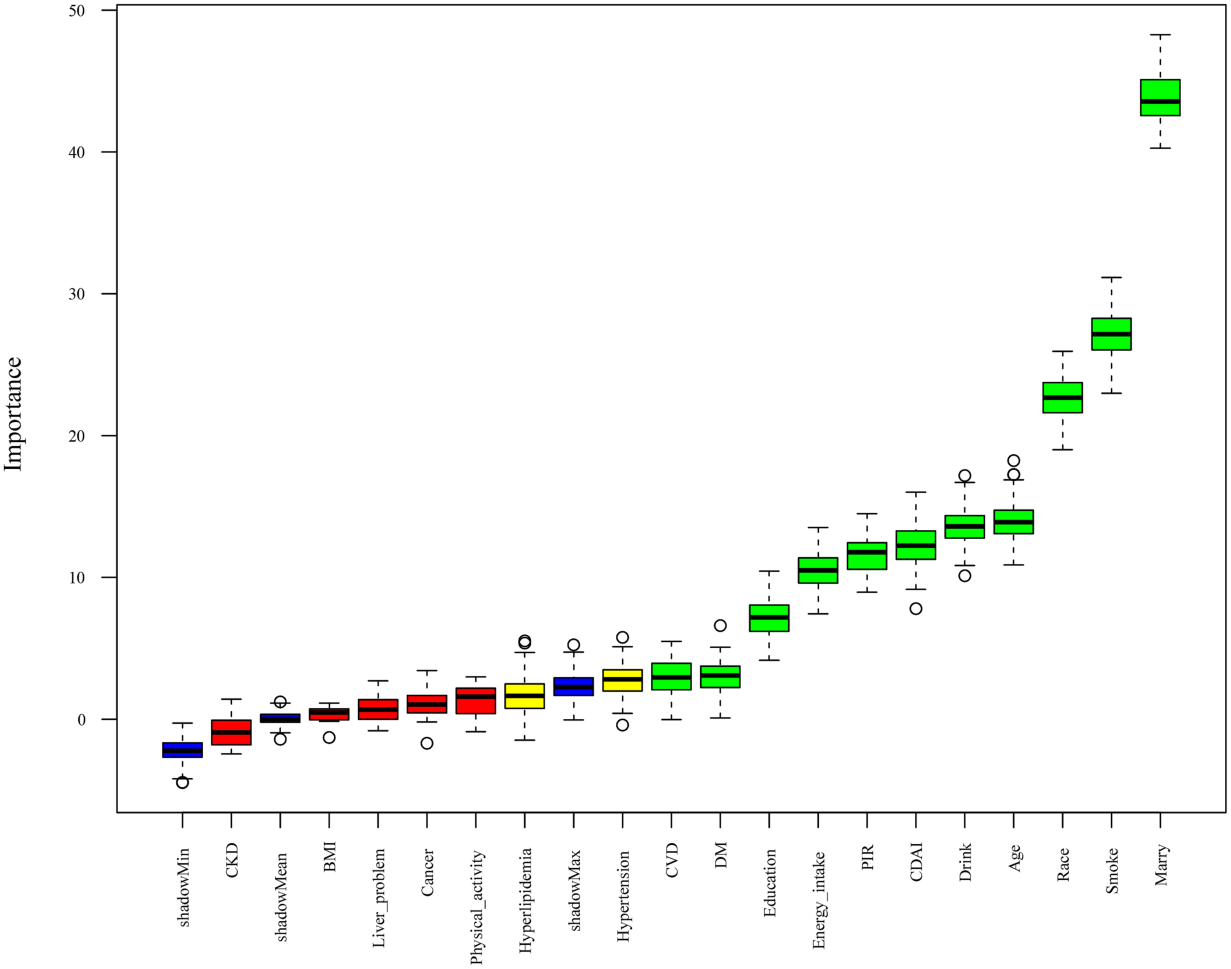
Boruta algorithm for selecting ML model variables. ML, machine learning; BMI, body mass index; CVD, cardiovascular disease; CKD, chronic kidney disease; DM, diabetes mellitus; PIR, poverty-income ratio; CDAI, composite dietary antioxidant index.

**Figure 4:**
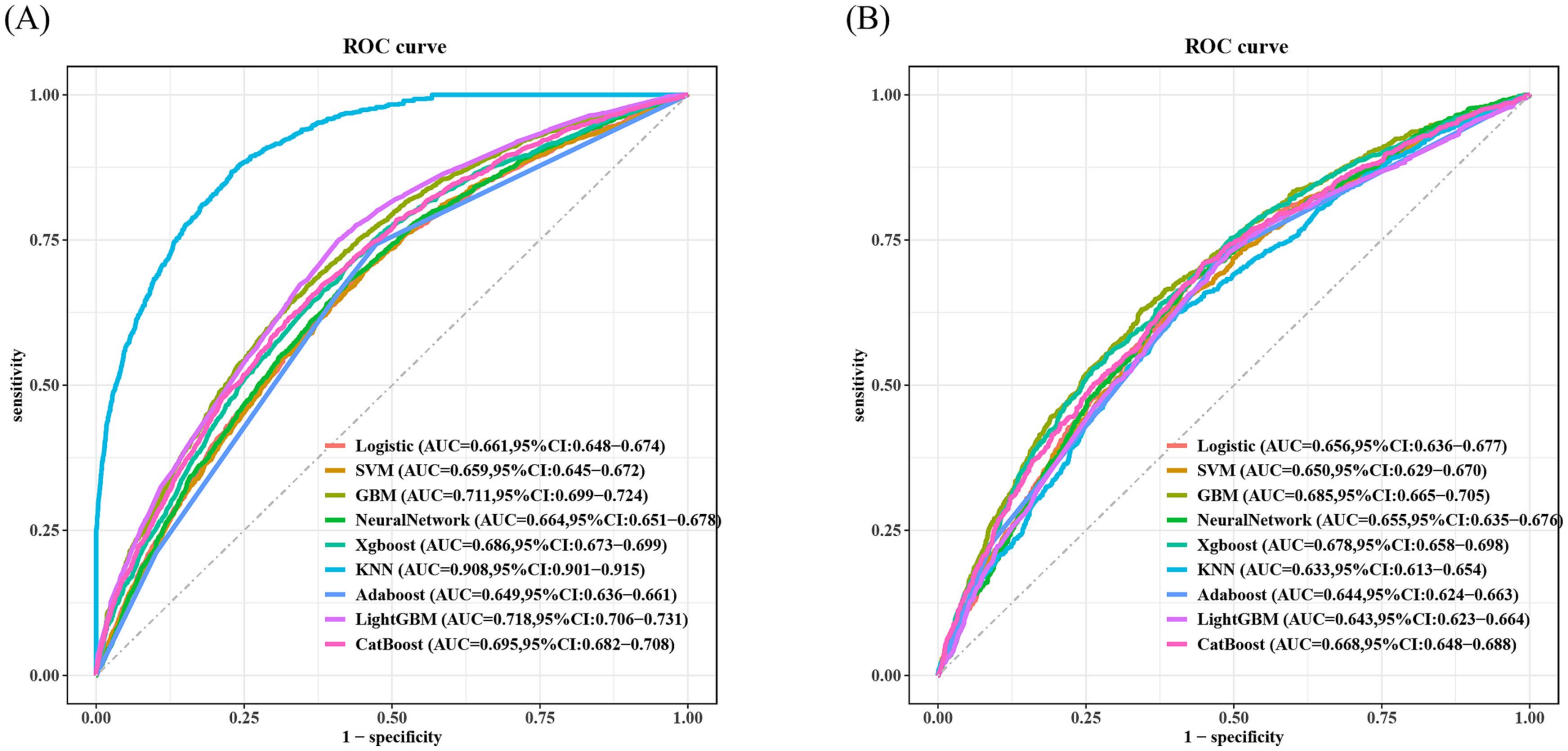
ROC curves in the training set and testing set. **(A)** ROC curves of the training set. **(B)** ROC curves of the testing set. ROC, receiver operating characteristic curve; AUC, Area Under Curve.

As shown in Figure 3, in this study, the Boruta algorithm was used for feature screening of 17 variables. After screening, 12 effective variables were determined for subsequent model prediction, including marital status, smoking, race, age, alcohol consumption, CDAI, PIR, dietary energy, educational level, diabetes, CVD, and hypertension. Figures 4A,B respectively show the ROC curves of the nine machine learning models on the training set and the test set. In addition, the calibration curves and DCA curves of these nine machine learning models were also analyzed to evaluate the prediction accuracy and clinical value of the models (Supplementary Figures 3, 4). In the test set, compared with the other eight models, the GBM model showed the best prediction performance (AUC = 0.685) (Figure 4B). Therefore, in the subsequent analysis, SHAP was used to interpret the GBM model.

### 3.6 Model decision of SHAP

**Figure 5:**
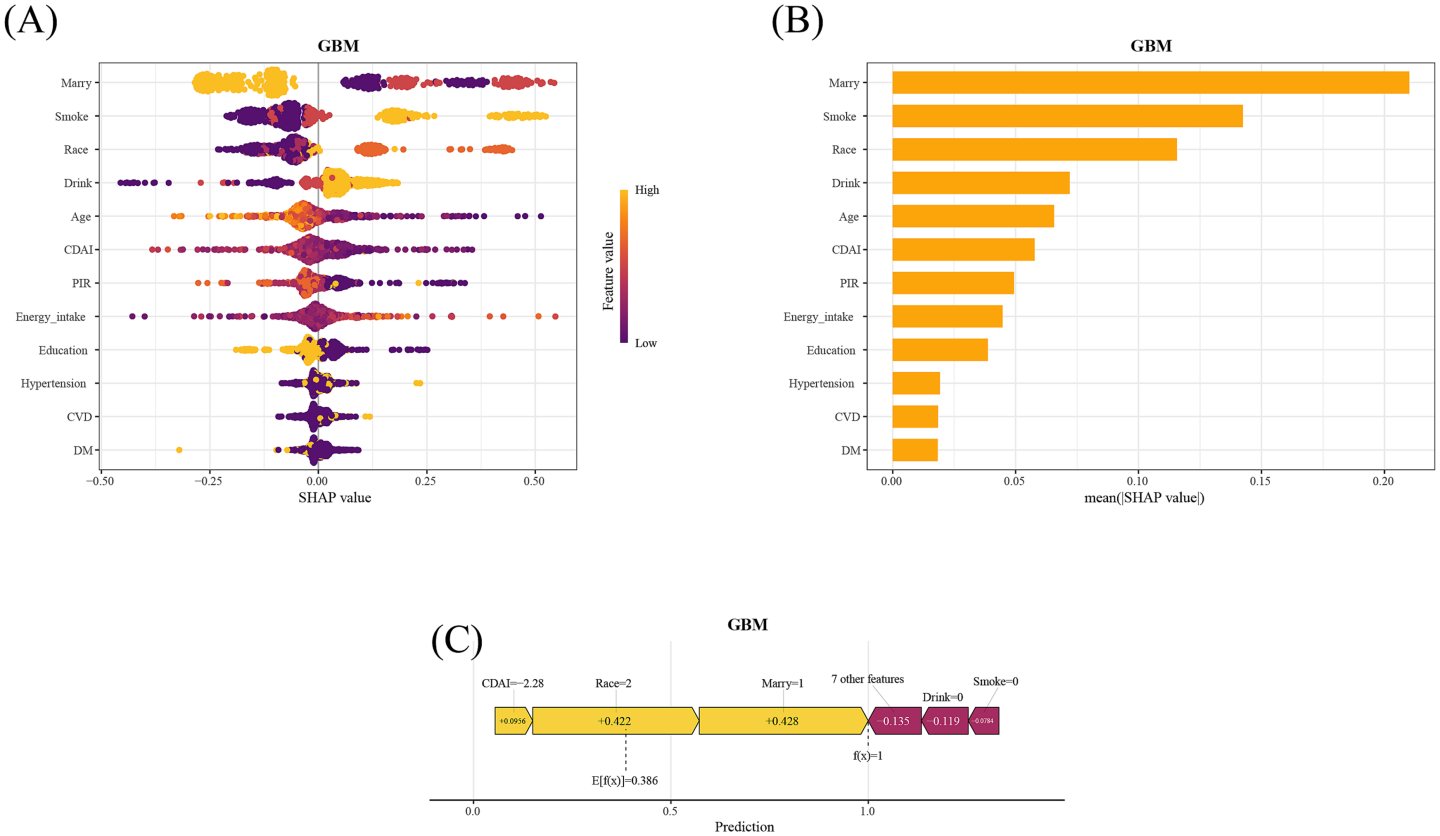
SHAP diagram for interprete GBM model. **(A)** SHAP honeycomb diagram of the GBM model. **(B)** SHAP value ranking of the variables in the model. **(C)** SHAP force plot for the twelfth sample in the study population. SHAP, SHapley Additive exPlanations; GBM, Gradient Boosting Machine; CDAI, composite dietary antioxidant index; DM, diabetes mellitus; CVD, cardiovascular disease; PIR, poverty-income ratio.

The SHAP algorithm was used to evaluate the importance of each feature variable in the GBM model and its contribution to the model’s prediction (Figures 5A,B). Among the 12 predictive variables included in the model, marital status, smoking, race, alcohol consumption, age, and CDAI ranked among the top six. As shown in Figure 5C, we used SHAP force plot to visualize the direction and degree of the influence of different variable features of the 12th participant in the study population on the final predicted value. As shown in the figure, the actual value [f(x)] of this participant was greater than the expected value (E[f(x)]), so this was a positive sample with HPV infection. The lower CDAI value (−2.28) of this participant increased the SHAP value by 0.0956, which indicates that a lower CDAI tends to explain the participant as having HPV infection, further enhancing the reliability of the negative correlation between CDAI and HPV infection.

## 4 Discussion

This study analyzed the data from NHANES 2003–2016. Firstly, we investigated the association between CDAI and HPV infection through multivariate logistic regression and found that CDAI, presenting an L-shaped pattern, reduced the likelihood of HPV infection, and this association was stronger among married people. Secondly, we also investigated the individual and combined associations between the six sub-components of CDAI and HPV infection, and found that vitamin E was the strongest factor influencing HPV infection. In addition, among the nine ML models, the GBM model showed the best predictive performance, with a ROC of 0.685 after 10-fold cross-validation, indicating the accuracy and reliability of the GBM model with 12 variables in predicting HPV infection. Finally, we used the SHAP algorithm to visually interpret the GBM model and found that a decrease in CDAI tended to classify an individual as a positive sample, emphasizing its important clinical value in predicting HPV risk.

Currently, the conclusions of existing studies on HPV infection and antioxidant diets are not entirely consistent. Only one study from Italy has investigated the relationship between CDAI and HPV infection. In this cross-sectional study, among 251 Italian women with normal cervical cytology, it was found that participants in the highest tertile group of CDAI had a 61% lower probability of being HPV-positive compared to those in the lowest tertile group (5). This is consistent with our findings. Our study showed that participants in the highest quartile group had a 23% lower prevalence of HPV infection compared to those in the lowest quartile group. Several studies have investigated the relationship between individual antioxidants and HPV infection. In a large cross-sectional study, Huang et al. (13) found a U-shaped association between dietary vitamin A and the risk of HPV, that is, both too low and too high levels of vitamin A were risk factors for HPV. Zheng et al. (14) also found a U-shaped association between vitamin C and the risk of HPV. Our study complements this. In the BKMR model, the intakes of dietary vitamin A and vitamin C did not seem to affect the likelihood of HPV infection. Vitamin E appears to be a more promising protective factor. Some studies have shown that dietary vitamin E intake has a negative linear relationship with both high-risk and low-risk HPV infections (15). A cohort study in Brazil showed that the serum vitamin E level may have a protective effect on the persistence of non-carcinogenic HPV (16). Our WQS model and BKMR model consistently showed the powerful effect of vitamin E in reducing HPV infection. In the WQS model, vitamin E showed the largest weight value; in the BKMR model, when the other five antioxidants were fixed at the 25th, 50th, and 75th percentile values, vitamin E was significantly negatively correlated with HPV infection. In addition, a multicenter study pointed out that women in the highest quartile of plasma cis- and total *β*-carotene had approximately a 50% reduced risk of HPV infection (17). A randomized controlled trial (RCT) involving 80 subjects showed that oral zinc sulfate supplementation for 3 months could increase the clearance rate of HPV and promote the regression of pre-existing cervical lesions (18). A study in western China showed that a serum selenium content of ≥ 0.02 mg/kg was a protective factor against HPV infection (19). In our single-exposure model of BKMR, the effects of β-carotene, zinc, and selenium were rather limited.

In terms of the mechanisms, firstly, an antioxidant diet can enhance the body’s antioxidant defense system. HPV infection can trigger intracellular oxidative stress, damaging cellular DNA, proteins, and lipids, and affecting the normal functions of cells and the immune response (25). CDAI encompasses various antioxidant components such as zinc, selenium, vitamin A, C, E, and carotenoids. These components can scavenge free radicals in the body and reduce oxidative stress damage (26). Secondly, antioxidants have an immunomodulatory effect. Reactive oxygen species (ROS) are considered to have an impact on the function and proliferation of T cells. A low concentration of ROS in T cells is a prerequisite for cell survival, while an increase in ROS accumulation can lead to apoptosis/necrosis (27). Many antioxidants are involved in the development, differentiation, and functional regulation of immune cells (27). Antioxidants such as vitamin E and *β*-carotene can enhance the functions of immune cells (such as natural killer cells, T cells, and B cells), promote their proliferation and differentiation, and improve the immune response (28, 29). In particular, vitamin E has been shown to enhance the immune response in animal and human models and confer protection against a variety of infectious diseases (30). Thirdly, antioxidants may interfere with the life cycle of HPV. After HPV infects host cells, it needs to utilize the metabolic and signaling pathways of host cells to complete replication, transcription, and assembly (31, 32). Antioxidants can regulate intracellular signaling pathways and affect the expression of HPV viral proteins and the assembly of viral particles. For example, vitamin E can inhibit viral protein synthesis, and carotenoids can interfere with the binding of the virus to cell receptors, hindering the HPV infection process (33, 34). Fourthly, antioxidants can promote cell repair and regeneration. HPV infection can damage cells, and antioxidants can promote cell repair and regeneration. Zinc is involved in the synthesis of DNA repair enzymes, which helps in the repair of damaged cellular DNA (35); vitamins A, C, etc. provide the raw materials and energy for cell repair and regeneration. When the CDAI is high, cells can more quickly and effectively repair the damage caused by HPV infection, reducing the risk of persistent infection. Finally, antioxidants can also regulate the inflammatory response. HPV infection often triggers an inflammatory response. Excessive inflammation can damage tissues and cells, affect immune function, and facilitate the persistent infection of the virus (36). Antioxidants can regulate the inflammatory response, inhibit the release of pro-inflammatory cytokines, and enhance the activity of anti-inflammatory cytokines (37).

Our study indicates that vitamin E is the most influential factor affecting HPV infection. HPV viruses are detected in 93% of invasive cervical cancer cases, and immune function serves as a critical defense against HPV invasion (38). The vitamin E content in immune cells is often significantly higher than that in other cells. As a fat-soluble antioxidant, vitamin E helps reduce free radical production and prevent lipid peroxidation, thereby protecting immune cells from oxidative damage (15, 30, 39). It also regulates immune cell functions by modulating signaling pathways, such as inhibiting inflammation-related pathways like NF-κB, PKC, and p38 MAPK (30). Additionally, prostaglandin E2 (PGE2) suppresses T-cell activation and proliferation by inhibiting IL-2 production and transferrin receptor expression (40). Vitamin E reduces PGE2 synthesis by inhibiting the activity of cyclooxygenase-2 (COX-2). For example, studies have shown that vitamin E supplementation can decrease COX activity by 60% (41). Moreover, vitamin E metabolites such as long-chain carboxyflavanols (LCMs) have been confirmed as potent COX inhibitors, with 13′-carboxyflavanol demonstrating particularly significant inhibitory effects on both COX-1 and COX-2 (42).

This study found that the effect of CDAI in reducing HPV infection is stronger in people who are married or have a partner. We speculate that this may be related to the following factors. Firstly, people who are married or have a partner often have a more regular lifestyle, with relatively fixed dietary and daily routine habits. For example, partners can supervise each other’s diet, increase the intake of foods rich in antioxidants, raise the CDAI level, and enhance the body’s ability to resist HPV infection. A regular daily routine can ensure the normal operation of the immune system, allowing antioxidants to function better and reducing the risk of infection (43, 44). Secondly, a stable partnership may have a positive impact on the immune system (45). The emotional support in an intimate relationship can reduce the secretion of stress hormones and prevent the suppression of the immune system (46). At this time, the antioxidant components in CDAI can more effectively regulate the function of immune cells, enhance immune surveillance, and the ability to eliminate HPV viruses, making the effect of CDAI in reducing HPV infection more significant. Thirdly, the sexual behavior of married people or those with partners may be more standardized, reducing the occurrence of high-risk sexual behaviors and lowering the risk of HPV exposure. In this situation, the protective effect of CDAI is more likely to be highlighted. Partners may remind each other to undergo HPV-related screenings, which is conducive to the early detection and intervention of infections, further strengthening the effect of CDAI in reducing infections. Finally, having a partner can bring a sense of psychological security and belonging, alleviating feelings of loneliness and anxiety (47). A good psychological state can promote the balance of the body’s endocrine system, creating a favorable internal environment for CDAI to play its role, enhancing the body’s resistance to HPV, and strengthening the effect of CDAI in reducing infections.

## 5 Advantages and limitations

Our study has the following advantages. Firstly, this is the first study to explore the individual and combined associations between the intakes of dietary vitamin A, vitamin C, vitamin E, zinc, selenium, and carotenoids and HPV infection based on a large sample population in the United States. Secondly, our study emphasizes the important value of dietary antioxidants, especially vitamin E, in the prevention of HPV infection. Finally, this study also constructed a predictive model, highlighting the accuracy of the GBM model in predicting HPV infection; and emphasizing the importance of controllable factors such as smoking, alcohol consumption, and dietary antioxidants in the prevention of HPV infection.

It is undeniable that this study has certain limitations. Firstly, the predictive model constructed in this study is based on cross-sectional data, a characteristic that determines that model predictions cannot establish causal links. These results still need to be further validated by future longitudinal studies, combined with biomarker detection (such as oxidative stress indicators) and interventional trials (such as randomized controlled trials of antioxidant diets), so as to further clarify the causal pathway and temporal dynamics between dietary antioxidants and HPV infection. Secondly, all the data in this study are from the NHANES database, which mainly reflects the situation of the US population. For populations in other countries, its applicability may be poor, and it is difficult to directly promote and apply it. Thirdly, the construction of CDAI depends on the subjective recollection and expression of participants, and there is a high possibility of recall bias in this process, which affects the accuracy of the research results. Fourthly, although the GBM model performed best among various machine learning methods, its AUC of 0.685 indicates that the model’s discriminative ability remains moderate. Specifically, the model may have insufficient capability to identify high-risk populations due to the lack of key predictors such as immunological function indicators (e.g., CD4^+^ T cell counts). Additionally, the moderate AUC value reflects limited applicability of the model in clinical settings. Without further optimization or integration of multi-dimensional variables, its predictive results should be interpreted in conjunction with clinical evaluations and laboratory test results. Finally, the ML model developed this time has not been externally validated, which greatly limits its promotion and use among different populations. In view of this, future studies can consider conducting large-scale, prospective, and multi-center cohort studies, or using Mendelian randomization methods to verify the relevant causal relationships and promote the further development of research in this field.

## 6 Conclusion

This study found that among adult women in the United States, an antioxidant diet, especially an increased intake of vitamin E, is significantly negatively correlated with HPV infection. The GBM model with 12 features can effectively predict the occurrence of HPV infection, and CDAI is an important factor in the model. In conclusion, increasing the intake of dietary antioxidants, especially vitamin E, may be crucial for the prevention of HPV infection.

## Data Availability

Publicly available datasets were analyzed in this study. This data can be found at: https://wwwn.cdc.gov/nchs/nhanes/.

## Glossary

Adaboost: Adaptive Boosting
AUC: Area Under Curve
BKMR: Bayesian kernel machine regression
BMI: Body mass index
CatBoost: Categorical Boosting
CDAI: composite dietary antioxidant index
CKD: chronic kidney disease
COX-2: cyclooxygenase-2
CVD: cardiovascular disease
DCA: decision curve analysis
DM: Diabetes mellitus
GBM: Gradient Boosting Machine
HPV: Human Papillomavirus
KNN: K-Nearest Neighbors
LCMs: long-chain carboxyflavanols
LightGBM: Light Gradient Boosting Machine
MCMC: Markov Chain Monte Carlo
ML: machine learning
NHANES: National Health and Nutrition Examination Survey
OR: odds ratio
PCR: polymerase chain reaction
PIR: poverty-income ratio
PGE2: prostaglandin E2
RCS: restricted cubic spline
RCT: randomized controlled trial
ROC: receiver operating characteristic curve
ROS: oxygen species
SD: standard deviation
SHAP: SHapley Additive exPlanations
SVM: Support Vector Machines
WQS: weighted quantile sum
Xgboost: eXtreme Gradient Boosting
