# Supplementary material for: Composite dietary antioxidant index and HPV infection from single and mixed associations to SHAP-interpreted machine learning predictions
Source: Front Nutr. 2025 Jul 31;12:1619742. doi: 10.3389/fnut.2025.1619742 (PMC12350107; doi:10.3389/fnut.2025.1619742)
Supplement: Supplementary file 1 [file Table_1.docx]

Composite dietary antioxidant index and HPV infection from Single and mixed associations to SHAP-interpreted machine learning predictions

# Supplementary Figures

## Supplementary Figure 1: Flow chart for inclusion of participants


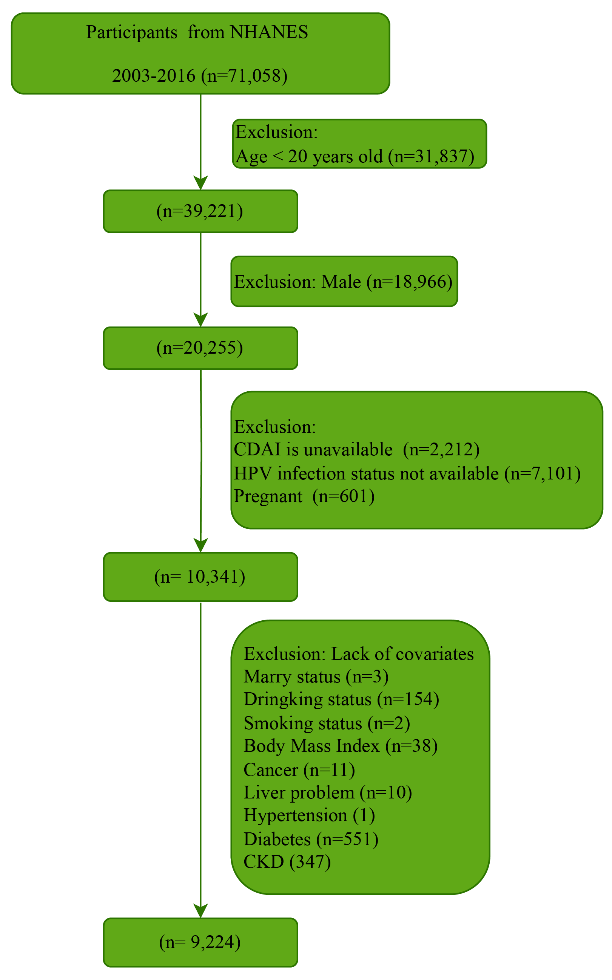


Notes: CDAI, composite dietary antioxidant index; HPV, human papillomavirus; BMI, body mass index; CVD, cardiovascular disease; CKD, chronic kidney disease; DM, diabetes mellitus.

## Supplementary Figure 2: Subgroup analysis and effect modification test of CDAI with HPV infection


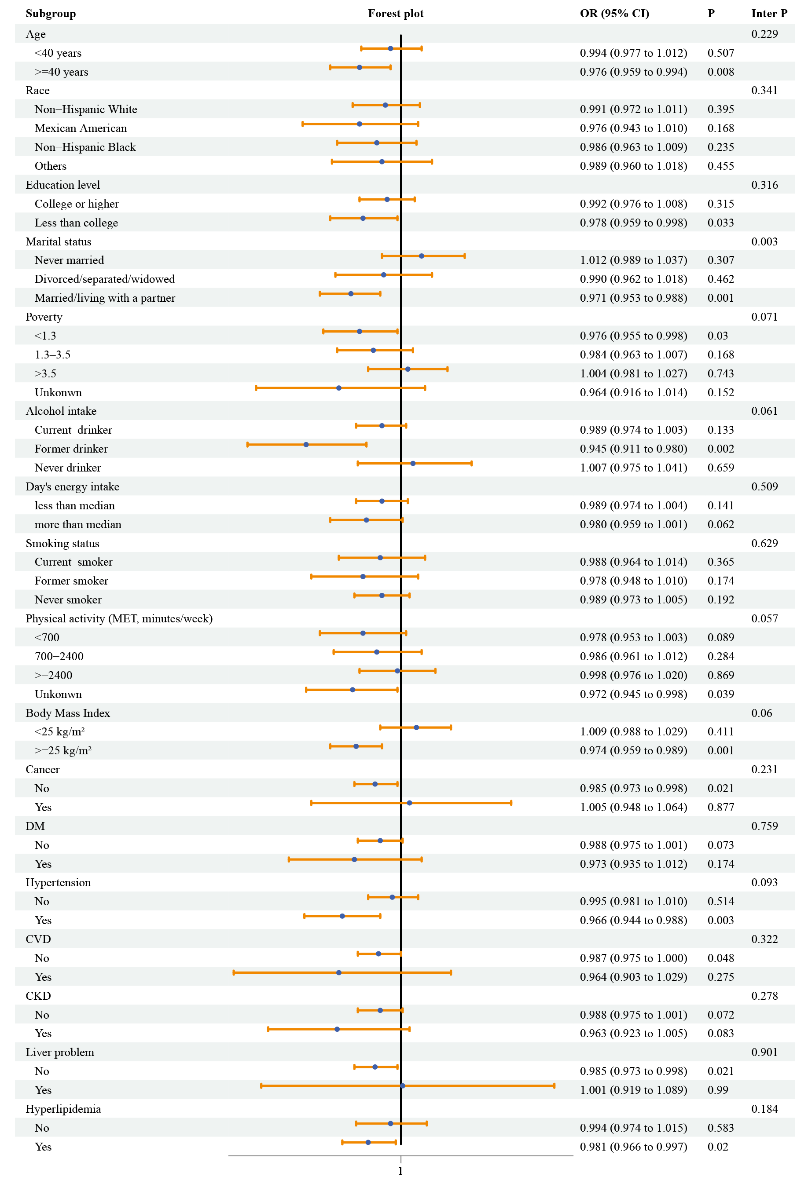


Notes: Models were adjusted for all covariates other than stratification variables. The significance of the interaction effect was determined by likelihood ratio test. CDAI, composite dietary antioxidant index; HPV, human papillomavirus; BMI, body mass index; CVD, cardiovascular disease; CKD, chronic kidney disease; DM, diabetes mellitus; OR, odds ratio; CI, confidence interval; inter P, P for interaction.

## Supplementary Figure 3: Calibration curves in training and testing set.


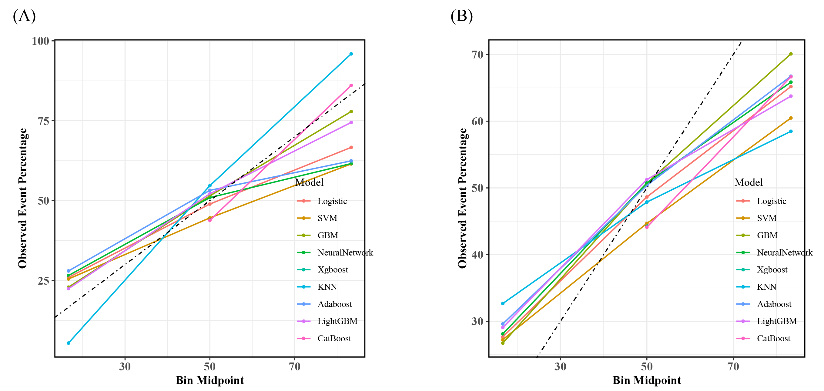


Notes: (A): Calibration curves in training set; (B): Calibration curves in testing set. Logistic, Logistic Regression; SVM, Support Vector Machines; GBM, Gradient Boosting Machine; Xgboost, eXtreme Gradient Boosting; KNN, K-Nearest Neighbors; Adaboost, Adaptive Boosting; LightGBM, Light Gradient Boosting Machine; CatBoost, Categorical Boosting.

## Supplementary Figure 4: DCA curves in training and testing set.


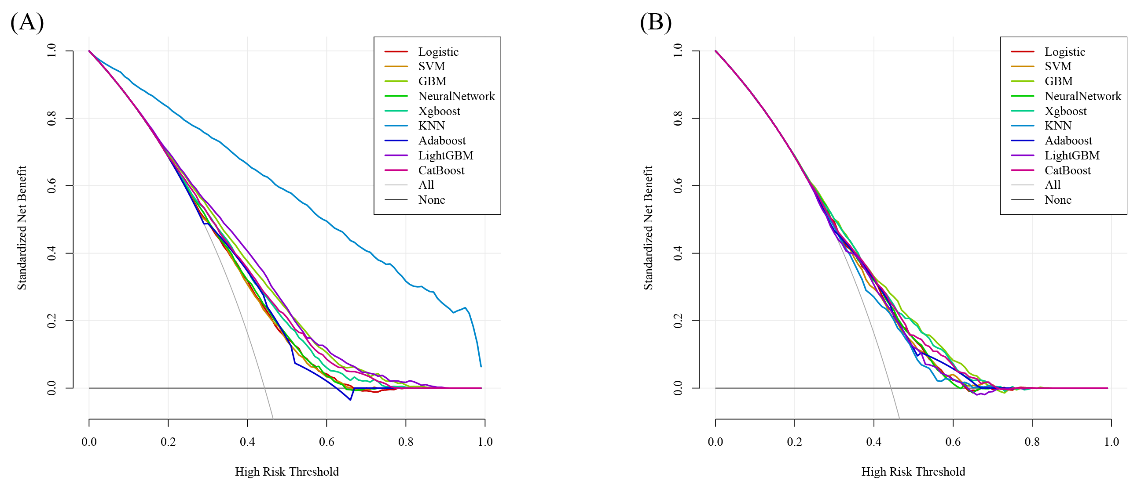


Notes: (A): DCA curves in training set; (B): DCA curves in testing set. DCA, decision curve analysis; Logistic, Logistic Regression; SVM, Support Vector Machines; GBM, Gradient Boosting Machine; Xgboost, eXtreme Gradient Boosting; KNN, K-Nearest Neighbors; Adaboost, Adaptive Boosting; LightGBM, Light Gradient Boosting Machine; CatBoost, Categorical Boosting.

# Supplementary Tables

## Supplementary Table 1. Information of Covariates.

| **Covariables** | | |
| --- | --- | --- |
| Demographic, Socioeconomic | Lifestyle | Health-related characteristics |
| Age (continuous) | Drinking status | Hyperlipidemia |
| Race | Former drinker | No |
| Mexican American | Never drinked | Yes |
| Non-Hispanic Black | Current drinker | Hypertension |
| Non-Hispanic White | Smoking status | No |
| Others | Never smoked | Yes |
| Educational level | Former smoker | DM |
| No college | Current smoker | DM |
| College or equivalent | Physical activity (MET), minutes/week | IFG |
| Marital status | <700 | IGT |
| Divorced or separated or widowed | 700-2400 | No |
| Never married | >=2400 | Cancer |
| Already married or cohabitation | Not report | No |
| Poverty to income ratio | Day's energy intake (continuous) | Yes |
| <1.3 | Body mass index | CVD, n (%) |
| 1.3–3.5 | <25 kg/m^2^ | No |
| >3.5  Unvailable | >=25 kg/m^2^ | Yes |
|  |  | CKD, n (%) |
|  |  | No |
|  |  | Liver problem  No  Yes |

**Abbreviations:** CDAI, composite dietary antioxidant index; HPV, human papillomavirus; BMI, body mass index; CVD, cardiovascular disease; CKD, chronic kidney disease; DM, diabetes mellitus.

## Supplementary Table 2: Sensitivity analysis for the association between CDAI and HPV infection.

|  | Model 0 | | Model 1 | | Model 2 | | Model 3 | |
| --- | --- | --- | --- | --- | --- | --- | --- | --- |
|  | OR (95% CI) | *P* | OR (95% CI) | *P* | OR (95% CI) | *P* | OR (95% CI) | *P* |
| **Sensitivity 1** |  |  |  |  |  |  |  |  |
| CDAI (continuous) | 0.97(0.96,0.98) | <0.0001 | 0.98(0.97,0.99) | 0.002 | 0.98(0.97,0.99) | 0.01 | 0.98(0.97,1.00) | 0.01 |
| CDAI (classify) |  |  |  |  |  |  |  |  |
| quartile 1 | ref |  | ref |  | ref |  | ref |  |
| quartile 2 | 0.83(0.74,0.94) | 0.002 | 0.92(0.82,1.04) | 0.18 | 0.90(0.80,1.03) | 0.12 | 0.92(0.81,1.04) | 0.20 |
| quartile 3 | 0.69(0.61,0.77) | <0.0001 | 0.79(0.70,0.89) | <0.001 | 0.77(0.67,0.88) | <0.001 | 0.78(0.68,0.89) | <0.001 |
| quartile 4 | 0.71(0.63,0.80) | <0.0001 | 0.82(0.72,0.92) | 0.001 | 0.77(0.66,0.90) | 0.001 | 0.79(0.67,0.93) | 0.004 |
|  |  |  |  |  |  |  |  |  |
| **Sensitivity 2** |  |  |  |  |  |  |  |  |
| CDAI (continuous) | 0.97(0.96,0.98) | <0.0001 | 0.98(0.97,0.99) | 0.002 | 0.98(0.97,0.99) | 0.01 | 0.98(0.97,0.99) | 0.01 |
| CDAI (classify) |  |  |  |  |  |  |  |  |
| quartile 1 | ref |  | ref |  | ref |  | ref |  |
| quartile 2 | 0.83(0.74,0.94) | 0.002 | 0.92(0.82,1.04) | 0.18 | 0.90(0.80,1.03) | 0.12 | 0.91(0.80,1.04) | 0.16 |
| quartile 3 | 0.69(0.61,0.77) | <0.0001 | 0.79(0.70,0.89) | <0.001 | 0.77(0.67,0.88) | <0.001 | 0.78(0.68,0.89) | <0.001 |
| quartile 4 | 0.71(0.63,0.80) | <0.0001 | 0.82(0.72,0.92) | 0.001 | 0.77(0.66,0.90) | 0.001 | 0.77(0.66,0.90) | 0.001 |
|  |  |  |  |  |  |  |  |  |
| **Sensitivity 3** |  |  |  |  |  |  |  |  |
| CDAI (continuous) | 0.97(0.96,0.98) | <0.0001 | 0.98(0.97,0.99) | 0.002 | 0.98(0.97,0.99) | 0.01 | 0.98(0.97,1.00) | 0.01 |
| CDAI (classify) |  |  |  |  |  |  |  |  |
| quartile 1 | ref |  | ref |  | ref |  | ref |  |
| quartile 2 | 0.83(0.74,0.94) | 0.002 | 0.92(0.82,1.04) | 0.18 | 0.90(0.80,1.03) | 0.12 | 0.92(0.81,1.05) | 0.21 |
| quartile 3 | 0.69(0.61,0.77) | <0.0001 | 0.79(0.70,0.89) | <0.001 | 0.77(0.67,0.88) | <0.001 | 0.78(0.68,0.90) | <0.001 |
| quartile 4 | 0.71(0.63,0.80) | <0.0001 | 0.82(0.72,0.92) | 0.001 | 0.77(0.66,0.90) | 0.001 | 0.79(0.67,0.93) | 0.004 |

**Notes:**

Model 0: No covariate was adjusted.

Model 1: Adjusted for age, race, education attainment, marital status, and poverty-income ratio.

Model 2: Further adjusted for smoking, drinking status, BMI, physical activity, and energy intake based on Model 1.

Model 3: In sensitivity analysis 1, it was further adjusted for hyperlipidemia, hypertension, DM, liver diseases, cancer, CVD, CKD, number of sexual partners, and condom use; In sensitivity analysis 2, it was further adjusted for hyperlipidemia, hypertension, DM, liver diseases, cancer, CVD, CKD, and HPV vaccination; In sensitivity analysis 3, it was further adjusted for hyperlipidemia, hypertension, DM, liver diseases, cancer, CVD, CKD, number of sexual partners, condom use, and HPV vaccination.

**Abbreviations:** CDAI, composite dietary antioxidant index; HPV, human papillomavirus; BMI, body mass index; CVD, cardiovascular disease; CKD, chronic kidney disease; DM, diabetes mellitus; OR, odds ratio; CI, confidence interval.

## Supplementary Table 3: Association between WQS index of composite antioxidant diet and HPV infection

| Outcome | OR | 95% CI | *P* |
| --- | --- | --- | --- |
| HPV infection | 0.78 | (0.71, 0.86) | < 0.0001 |

**Notes:** HPV, human papillomavirus; WQS, weighted quantile sum regression; OR, odds ratio; CI, confidence interval.

## Supplementary Table 4: PIP values of 6 dietary antioxidants in BKMR

| Variables | PIP |
| --- | --- |
| Vitamin A | 0 |
| Vitamin C | 0 |
| Vitamin E | 0.68 |
| Zinc | 0.28 |
| Selenium | 0.50 |
| Carotenoid | 0 |

**Notes:** PIP, Posterior Inclusion Probability; BKMR, Bayesian kernel machine regression.
